# Validity of self-assessment of hallux valgus using the Manchester scale

**DOI:** 10.1186/1471-2474-11-215

**Published:** 2010-09-20

**Authors:** Hylton B Menz, Mohammad R Fotoohabadi, Elin Wee, Martin J Spink

**Affiliations:** 1 Musculoskeletal Research Centre, Faculty of Health Sciences, La Trobe University, Bundoora, Victoria, 3086, Australia

## Abstract

**Background:**

Hallux valgus (HV) is a common condition involving the progressive subluxation of the first metatarsophalangeal joint due to lateral deviation of the hallux and medial deviation of the first metatarsal. The objective of this study was to evaluate the re-test reliability and validity of self-assessment of HV using a simple clinical screening tool involving four standardised photographs (the Manchester scale), in order to determine whether this tool could be used for postal surveys of the condition.

**Methods:**

HV was assessed with the Manchester scale in 138 people aged 65 to 93 years of age (102 women and 36 men) as part of a larger randomised controlled trial. At the six month follow-up assessment, HV was reassessed to determine re-test reliability, and participants were asked to self-assess their degree of HV independent of the examiners. Associations between (i) baseline and follow-up assessments of the examiners and (ii) participant and examiner assessments were performed using weighted kappa statistics. Analyses were then repeated after HV was dichotomised as present or absent using unweighted kappa, and sensitivity and specificity of self-assessment of HV was determined.

**Results:**

Re-test reliability of the examiners was substantial to almost perfect (weighted kappa = 0.78 to 0.90), and there was a substantial level of agreement between observations of the participants and the examiners (weighted kappa = 0.71 to 0.80). Overall, there was a slight tendency for participants to rate their HV as less severe than the examiners. When the Manchester scale scores were dichotomised, agreement was substantial to almost perfect for both re-test comparisons (kappa = 0.80 to 0.89) and substantial for comparisons between participants and examiners (kappa = 0.64 to 0.76). The sensitivity and specificity of self-assessment of HV using the dichotomous scale were 85 and 88%, respectively.

**Conclusions:**

The Manchester scale demonstrates high re-test reliability, and self-assessment scores obtained by participants are strongly associated with scores obtained by examiners. These findings indicate that the tool can be used with confidence in postal surveys to document the presence and severity of HV.

**Trial registration:**

ACTRN12608000065392

## Background

Hallux valgus (HV) is a common condition affecting the forefoot in which the first metatarsophalangeal joint is progressively subluxed due to the lateral deviation of the hallux and medial deviation of the first metatarsal [1]. The resultant deformity often leads to the development of a soft tissue and osseous prominence on the medial aspect of the first metatarsal head, commonly referred to as a "bunion" [2]. Prevalence estimates of HV range from 21 to 65% [3-9], with the largest study so far undertaken (involving 4,249 people aged over 30 years) reporting a prevalence of 28% [10]. HV has been shown to have a detrimental impact on health-related quality of life [11-14], and is associated with impaired gait [15] and balance [16] and an increased risk of falls [17,18] in older people. Surgical correction of HV is one of the most commonly-performed orthopaedic foot and ankle procedures [19,20].

HV is generally considered to be present when the angle formed by the bisections of the first metatarsal and the proximal phalanx obtained from foot radiographs is greater than 15 degrees [21,22]. However, because it is not always feasible or necessary to obtain radiographs to assess HV, several other approaches have been suggested, including goniometric assessment, measurement of forefoot girth, and the use of standardised photographs or line drawings [23-26]. The most developed of these tools are the Manchester scale [25] and a line drawing tool described by Roddy et al [26]. The Manchester scale consists of standardised photographs of feet with four grades of HV (none, mild, moderate and severe). Both re-test and inter-tester reliability of grading HV using the Manchester scale have been found to be excellent (kappa values of 0.77 and 0.86, respectively [25,27]). More recently, Roddy et al [26] developed an instrument consisting of five line drawings, each drawing illustrating a sequential increase in the HV angle of approximately 15 degrees. This tool has also been shown to have excellent re-test reliability (kappa = 0.82).

Although either of these tools can be used to provide accurate information regarding the presence and severity of HV, each tool has advantages and disadvantages. The key advantages of the Manchester scale are that the photographs represent real cases of HV selected by a consensus panel of podiatrists to represent the full spectrum of the deformity, and that scores documented using this tool have been shown to be highly correlated with angular measurements obtained from foot radiographs [28]. By comparison, the Roddy et al [26] tool uses stylised line drawings with hypothetical degrees of deformity, and has not yet been validated against radiographs. The key disadvantage of the Manchester scale is that it has not yet been validated as a self-assessment tool, thereby limiting its application to settings where trained observers are used to document the presence and severity of HV. Therefore, the primary objective of this study was to address this shortcoming by evaluating the level of agreement between trained clinical assessment and self-assessment of HV using the Manchester scale. A secondary objective was to evaluate re-test reliability of clinical observations of HV over a longer period than has been previously undertaken for this tool (i.e. six months compared to two weeks). In doing so, our aim was to determine whether the Manchester scale would be a suitable tool for self-assessment of HV in the context of a postal survey of foot disorders.

## Methods

### Participants

Participants were drawn from a larger randomised controlled trial investigating the efficacy of a podiatry intervention to prevent falls (Trial Registration Number: ACTRN12608000065392), the details of which are described elsewhere [29]. Briefly, community dwelling men and women aged 65 years and over were recruited by a mail-out letter from a database of people who were accessing podiatry services at the La Trobe University Health Sciences Clinic, Bundoora, Victoria, Australia as well as from advertisements placed in seniors newspapers and websites. Inclusion criteria included an elevated risk of falling and current foot pain. Exclusion criteria included Parkinson's disease (or other neurodegenerative disorders), lower limb amputation and cognitive impairment. The Human Ethics Committee of La Trobe University approved the study (ID: 07-118) and all participants provided written informed consent.

### Manchester scale assessment

At the baseline assessment, all participants were assessed for HV using the Manchester scale by one of two examiners - a physiotherapist with 22 years of general physiotherapy clinical experience (MF) and a physiotherapist with 10 years of general physiotherapy clinical experience (EW). Both examiners had been trained in the use of the tool by an experienced podiatrist (MJS) prior to commencement of the study, using a sample of 36 older people recruited to pilot the clinical assessments used in the randomised controlled trial [30]. This process involved independent assessments by the podiatrist and the two examiners, which was followed by a discussion in which any discrepancies in interpretation of the scale were resolved.

**Figure 1:**
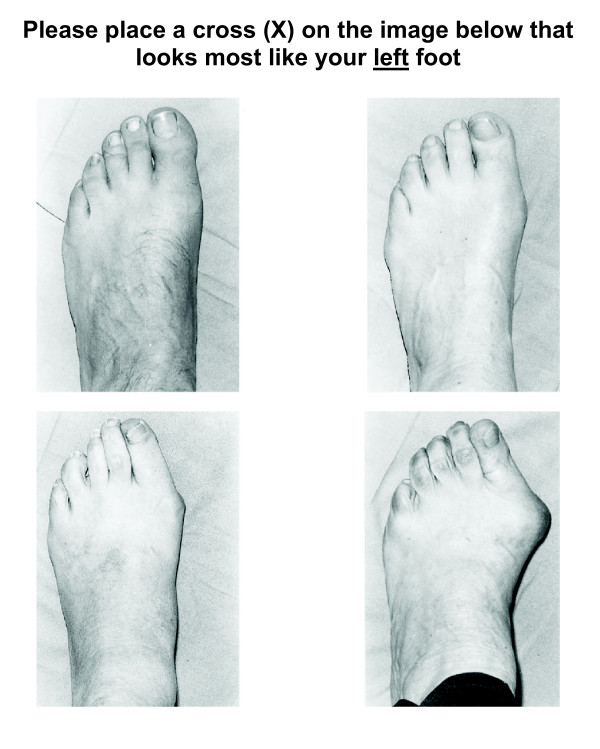
**Self-assessment of hallux valgus using the Manchester scale (left foot shown)**. Top left: no deformity (score = 0), top right: mild deformity (score = 1), bottom left: moderate deformity (score = 2), bottom right: severe deformity (score = 3). Figure adapted from Garrow et al [25].

At the six month follow-up assessment, the examiners repeated their assessment of HV without reference to their baseline scores. During the same session, the participants were then asked to independently assess their own feet. To do this, larger versions of the four photographs in the original Manchester scale publication [25] were printed on two sheets of A4 paper, with the images rotated to represent left feet and right feet on separate pages. Participants were instructed: "In this test, we would like you to compare your foot to the four pictures that are on the page. Whichever one of those four pictures you think most resemble your foot, we would like you to mark an × on the picture. There is no right or wrong answer, just whatever you think most closely resembles your foot". Participants were blinded to the examiners' assessments, and received no assistance from the examiners when completing their assessment. For all assessments, HV was documented as no deformity (score = 0), mild deformity (score = 1), moderate deformity (score = 2) or severe deformity (score = 3). See Figure 1.

### Statistical analysis

All analyses were performed using SPSS Statistics version 17.0 (SPSS Inc, Chicago, IL) and STATA version 8.2 (STATA Corp, College Station, TX). Statistical analysis was undertaken in three stages. Firstly, re-test reliability and agreement between HV severity scores obtained by the examiners and the participants was determined using percentage agreement in addition to weighted kappa (κ_w_), which is considered to be the most appropriate statistic to assess the level of agreement when the measurement scale is ordinal. In contrast to the ''standard'' κ described by Cohen [31], κ_w _also takes into account that the relative importance of disagreement between categories may not be the same for adjacent categories as it is for distant categories. For example, if one examiner documented HV as a score of 3 while the other scored it as a 2, the κ_w _approach would consider this to be less of an error compared to one examiner scoring a 0 and the other scoring a 3. The following quadratic assignment of weights described by Fleiss [32] was used:

$$w_{ij}=1-\frac{{\left(i-j\right)}^{2}}{{\left(k-1\right)}^{2}}$$

where *w *represents the weighting, *i *is the number of the row, *j *is the number of the column, and *k *is the total number of categories (in this case, four). The following benchmarks for interpretation of κ_w _scores were used: ≤ 0 = poor, 0.01 to 0.20 = slight, 0.21 to 0.40 = fair, 0.41 to 0.60 = moderate, 0.61 to 0.80 = substantial, and 0.81 to 1.00 = almost perfect [33]. To explore the level of disagreement between examiner and participant assessments, the frequency of disagreement types was determined, i.e. the number of occasions in which scores varied by a single category, 2 categories, and 3 categories. These analyses were performed for left feet, right feet, and with both feet combined.

Secondly, HV was dichotomised using the Manchester scale by merging the first two categories (i.e. scores of 0 or 1) to indicate that HV was absent, and merging the second two categories (i.e. scores of 2 or 3) to indicate that HV was present. This cut-off was based on our previous study where we found that the mean hallux abductus angle obtained from radiographs for participants with a Manchester scale score of 2 was approximately 15 degrees [28], which is the commonly accepted minimum value for the diagnosis of HV [21,22]. Re-test reliability and agreement between dichotomous scores obtained by the examiners and the participants was then determined using percentage agreement in addition to the standard (unweighted) kappa statistic (κ), with the same benchmarks for interpretation [33]. These analyses were also performed for left feet, right feet, and with both feet combined.

Thirdly, the sensitivity and specificity were calculated for the dichotomous self-assessment scores, using the examiners' dichotomous scores as the diagnostic "gold standard". This analysis was undertaken for both feet combined.

## Results

From the total sample of n = 305 recruited for the randomised controlled trial, the final 138 participants attending for their six month follow-up appointment formed the sample for this analysis. This group consisted of people aged 65 to 93 years of age (102 women and 36 men). Participant demographic characteristics and major self-reported medical conditions are shown in Table 1.

**Table 1:** Participant characteristics.

Characteristic	
Age (years) - mean (SD)	73.2 (5.8)
Height (cm) - mean (SD)	163.8 (8.1)
Weight (kg) - mean (SD)	78.9 (16.0)
Body mass index (kg/m^2^) - mean (SD)	29.3 (5.0)
Major medical conditions - n (%)	
Stroke	7 (5.1)
Diabetes	15 (10.9)
Heart disease	25 (18.1)
High blood pressure	73 (52.9)
Osteoarthritis	104 (75.4)

SD = standard deviation.

### Re-test reliability of HV assessment

The level of agreement between baseline and six month follow-up assessments of HV documented by examiners using the Manchester scale (i.e. re-test reliability) is shown in Table 2. Agreement was substantial to almost perfect (κ_w _between 0.78 and 0.90 and percentage agreement between 95.8 and 98.1%).

**Table 2:** Associations between baseline and 6 month follow-up assessments of hallux valgus using the Manchester scale (i.e. re-test reliability).

	**κ**_**w **_**(95% CI)**	% agreement
Left foot	0.88 (0.81 to 0.89)	98.1
Right foot	0.90 (0.89 to 0.91)	97.9
Both feet	0.78 (0.77 to 0.81)	95.8

κ_w _= weighted kappa statistic, CI = confidence interval.

### Agreement between examiner and participant assessment of HV

**Figure 2:**
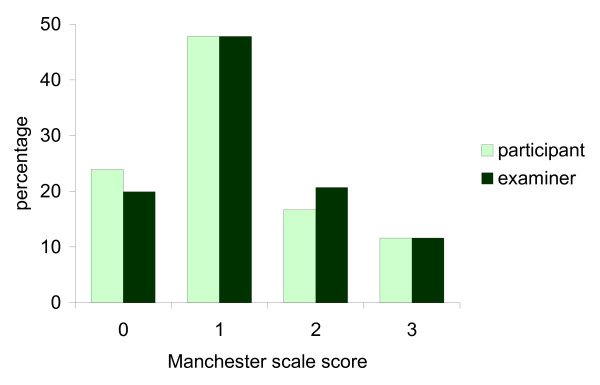
**Manchester scale scores obtained by participants and examiners (both feet combined)**.

The frequency of Manchester scale scores obtained by participants and examiners (for both feet combined) are shown in Figure 2. Overall, there was a slight tendency for participants to rate their HV as less severe than the examiners, as evidenced by a higher frequency of no deformity (0) scores and a lower frequency of moderate (2) scores. The level of agreement between examiner and participant assessments of HV using the Manchester scale is shown in Table 3. Agreement was substantial (κ_w _between 0.71 and 0.80 and percentage agreement between 95.1 and 96.1%). The frequencies of disagreement types between examiner and participant assessments are shown in Table 4, which indicates that most disagreements were of a magnitude of one, i.e. the examiner and the participant scores differed by only one category of HV severity. In no cases did the scores differ by three categories between examiners and participants.

**Table 3:** Associations between examiner and participant assessments of hallux valgus using the Manchester scale (i.e. validity).

	**κ**_**w **_**(95% CI)**	% agreement
Left foot	0.71 (0.62 to 0.73)	95.1
Right foot	0.80 (0.72 to 0.84)	96.1
Both feet	0.76 (0.75 to 0.79)	95.6

κ_w _= weighted kappa statistic, CI = confidence interval.

**Table 4:** Frequencies - n (%) of disagreement types between examiner and participant assessments of hallux valgus using the Manchester scale.

	No difference	Difference = 1	Difference = 2	Difference = 3
Left foot	83 (71.0)	53 (38.4)	2 (1.4)	0 (0)
Right foot	98 (60.1)	37 (26.8)	3 (2.1)	0 (0)
Both feet	181 (65.6)	90 (32.6)	5 (1.8)	0 (0)

### Dichotomous assessment of HV

The level of agreement for dichotomous grading of hallux valgus using the Manchester scale for both re-test and examiner versus participant comparisons is shown in Table 5. Agreement was substantial to almost perfect for re-test comparisons (κ between 0.80 and 0.89 and percentage agreement between 90.9 and 95.7%) and was substantial for comparisons of examiner and participant assessments (κ between 0.64 and 0.76 and percentage agreement between 85.5 and 89.1%). The diagnostic accuracy of the dichotomous self-assessment scores compared to the "gold standard" examiner assessment scores was high, with a sensitivity of 85% and a specificity of 88%.

**Table 5:** Re-test and examiner *vs *participant agreement of dichotomous grading of hallux valgus using the Manchester scale.

	Baseline *vs *follow-up (re-test reliability)		Examiner *vs *participant (validity)	
	**κ (95% CI)**	**% agreement**	**κ (95% CI)**	**% agreement**

Left foot	0.89 (0.81 to 0.98)	95.7	0.64 (0.49 to 0.78)	85.5
Right foot	0.87 (0.79 to 0.96)	94.2	0.76 (0.64 to 0.87)	89.1
Both feet	0.80 (0.72 to 0.87)	90.9	0.70 (0.61 to 0.79)	87.3

κ = kappa statistic, CI = confidence interval.

## Discussion

The objectives of this study were to evaluate the re-test reliability and validity of self-assessment of HV using a simple clinical screening tool involving four standardised photographs (the Manchester scale), in order to determine whether this tool could be used for postal surveys of foot disorders. The six month re-test reliability was very high, with κ_w _values between 0.78 and 0.90, and percentage agreement between 95.8 and 98.1%. Slightly lower re-test reliability (κ_w _= 0.77, percentage agreement = 84%) was reported by Menz et al [27] in three examiners assessing HV severity in 31 older people tested on two occasions, two weeks apart. This difference is likely to be due to the level of experience of the examiners. In the Menz et al [27] study, none of the three examiners had any experience in assessing foot disorders, whereas in the current study, the two examiners had recently been involved in undertaking foot assessments in a large number of participants involved in a clinical trial. The level of re-test reliability reported here for the Manchester scale is also similar to that reported for the line drawing scale described by Roddy et al [26], who evaluated the reliability of a single examiner assessing 25 participants on two occasions, three to six months apart. κ_w _values were 0.79 for the left foot, 0.84 for the right foot, and 0.82 when both feet were combined.

There was a high level of agreement between Manchester scale scores documented by the two examiners and those documented independently by the participants. Although there was a slight tendency for participants to rate their HV as less severe than the examiners, overall agreement was substantial (κ_w _values between 0.71 and 0.80 and percentage agreement between 95.1 and 96.1%), and when both feet were combined, 66% of the scores obtained were identical. Where disagreements were identified, the majority related to a difference of one category only. These findings compare favourably to results obtained with the five-level line drawing scale described by Roddy et al [26], who reported a lower overall κ_w _value of 0.45.

Although the Manchester scale is designed to categorise HV into four severity categories, in some situations it may be useful to have a dichotomous case definition. In this study, we developed a dichotomised case definition of HV by combining the first two categories to indicate that HV is absent, and combining the second two categories to indicate that HV is present. As it cannot be assumed that the reliability and validity of the four level scale is the same as the dichotomised scale, we also analysed the Manchester scale scores after they had been dichotomised. This made little difference to the results, with similarly high re-test reliability (κ values between 0.80 and 0.89) and agreement between the examiners and participants (κ values between 0.64 and 0.76). If it is assumed that the examiners' scores represent the "gold standard", self-assessments performed by the participants demonstrated excellent diagnostic accuracy, with a sensitivity of 85% and a specificity of 88%. In the Roddy et al [26] study, the dichotomous definition of HV using the line drawings exhibited similar re-test reliability (κ = 0.83), but lower participant-examiner agreement (κ = 0.55) and lower diagnostic accuracy (sensitivity of 75% and specificity of 82%).

The findings reported here suggest that the Manchester scale [25] may be a slightly more reliable and valid indicator of HV than the Roddy et al [26] line drawing tool, however a direct comparison of the two tools would be required to adequately ascertain this. Nevertheless, several differences between the tools are worthy of consideration in this context. Firstly, although the inclusion of five rather than four levels of severity in the Roddy et al [26] tool potentially allows for greater precision, this may also make the classification task slightly more difficult than the four options available in the Manchester scale, particularly for participants assessing their own feet. Secondly, there may be some additional visual assistance provided by the provision of photographs of real feet in the Manchester scale as opposed to line drawings. Thirdly, the two most severe depictions of HV in the Roddy et al [26] tool are accompanied by an under-riding second toe. Because the second toe may adopt a variety of postures in people with HV (including over-riding [34] and valgus [35] toe deformity), the depiction of the under-riding toe may create some confusion, despite the instructions requesting participants to focus only on their big toe. The potential distraction introduced by the inclusion of lesser toe deformity was identified by Garrow et al [25] when designing the Manchester scale, which resulted in the selection of the most severe HV photograph having no major deformity of the second toe.

The findings reported here need to be considered in the context of several study design limitations. Firstly, we were unable to assess the inter-examiner reliability of HV assessment in this study, as participants were drawn from a randomised controlled trial and all follow-up assessments needed to be conducted by the same examiner who conducted the baseline assessments. However, the inter-examiner reliability reported previously by Garrow et al [25] was very high (κ_w _values of 0.84 to 0.88). Secondly, the inclusion criteria for the larger trial from which this sample was obtained required participants to have current foot pain, which may have biased the sample towards having a higher than average prevalence of HV. Thirdly, participants' self-assessments were conducted in a clinical setting, and although the examiners did not provide any assistance, it is possible that the self-assessment scores may have been different if participants completed the task in their home environment. Finally, although the Manchester scale provides a useful overall indicator of the degree of angular deformity associated with HV, it is acknowledged that other factors, such as the degree of joint degeneration or sesamoid displacement, may be of equal or greater clinical importance in relation to the functional impact of the condition.

## Conclusions

Assessment of HV using the Manchester scale demonstrates high re-test reliability, and self-assessment scores obtained by participants are strongly associated with scores obtained by examiners, irrespective of whether the four-level classification or dichotomised scale are used. These findings indicate that the tool can be used with confidence in postal surveys to document the presence and severity of HV.

## Competing interests

The authors declare that they have no competing interests.

## Authors' contributions

HBM conceived the idea for the study, conducted the statistical analysis and drafted the manuscript. MF, EW and MJS collected and compiled the data and assisted with interpretation of the data and drafting of the manuscript. All authors read and approved the final manuscript.

## Pre-publication history

The pre-publication history for this paper can be accessed here:

http://www.biomedcentral.com/1471-2474/11/215/prepub
